# Estimation of genetic parameters and genetic trends for growth, reproductive, and survival traits of Bonga sheep using multi-variate animal model

**DOI:** 10.1371/journal.pone.0316129

**Published:** 2025-02-20

**Authors:** Metsafe Mamiru, Aberra Melesse, Aynalem Haile, Tesfaye Getachew

**Affiliations:** 1 Animal Research Directorate, Bonga Agricultural Research Center, Bonga, Ethiopia; 2 Department of Animal and Range Sciences, Hawassa University, Hawassa, Ethiopia; 3 The International Center for Agricultural Research in the Dry Areas (ICARDA), Addis Ababa, Ethiopia; Ain Shams University Faculty of Agriculture, EGYPT

## Abstract

The community-based breeding program was introduced to enhance the productivity of Bonga sheep. Data evaluating animals’ potential regarding key traits was gathered from 2009–2021. This study estimated covariance components and genetic parameters for growth (birth, weaning, and six-months weight), reproductive (lambing interval, annual reproductive rate, litter size at birth and weaning, litter weight at birth and weaning), and survival (0–3, 3–6, and 0–6 months) traits using a multivariate animal model. The Average Information Restricted Maximum Likelihood method in WOMBAT was used for growth and reproductive traits, while Derivative-free MUltivariate analysis was employed for survival traits. The results revealed that the additive heritability for growth, reproductive, and survival traits ranged from 0.15–0.26, 0.06–0.08, and 0.10–0.17, respectively. The variance ratio of maternal permanent environment for growth traits ranged from 0.07–0.25, while that of animal environment for reproductive traits ranged from 0.33–0.45. Maternal genetic heritability for birth, weaning, and six-months weights were 0.14, 0.19, and 0.19, respectively, while it ranged from 0.01–0.14 for reproductive traits. Additive genetic correlations among growth traits ranged from 0.04–0.79, and from 0.36–0.62 between growth and reproductive traits. Low to high additive genetic correlations (0.002–0.537) were recorded between growth and survival traits. Significant (P<0.001) and positive genetic trends were observed for most reproductive traits, though annual genetic trends were low. Moderate additive heritability for growth traits indicates potential for genetic improvement via selection, while the pronounced environmental impact on reproductive and survival traits suggests the need for improved management practices in addition to selection. The presence of positive genetic correlations of growth traits with reproductive and with survival traits suggests that selecting for improved weaning/six-month weight has the potential to enhance ewe productivity and lamb survival. This highlights the importance of considering both growth and reproductive/survival traits in breeding programs to achieve optimal outcomes in sheep production.

## 1. Introduction

Sheep are the second most important livestock species in Ethiopia next to cattle. According to the Central Statistical Agency (CSA), its total sheep population is estimated at 38 million [1]. Diverse breeds and ecotypes of sheep are distributed across Ethiopia, from the cool alpine climates of the mountains to the arid pastoral areas of the lowlands. Currently, nine genetically distinct breeds of sheep have been identified using both phenotypic and molecular methods [2]. This large sheep population and breed plays a crucial role in supporting the country’s economy and ensuring food security.

They play a vital role in enhancing the livelihoods of rural communities and contribute to the socio-economic well-being of those who raise them. Small ruminants contribute approximately 40% of the cash income for farm households, 19% of the total value of subsistence food from all livestock production, and 25% of the total domestic meat consumption [3]. Income from sheep enabled sheep producers to fulfill their daily home needs and other agricultural inputs like fertilizer in Adiyo district of Kafa zone, Ethiopia [4].

The primary objective of sheep farming in the Adiyo Kaka district is to generate income through the sale of live animals and to produce mutton [5]. To achieve these goals, it is essential to increase the number of lambs produced per ewe and improve the growth performance of the lambs. This requires improvements in ewe productivity, lamb growth, and lamb survival, as all of these have a direct impact on overall sheep productivity [6]. Therefore, selecting breeding stock with desirable traits such as high fertility, good mothering ability, and fast growth rates can improve the overall performance of the sheep flock.

Developing effective genetic improvement programs requires a comprehensive understanding of the genetic parameters and covariance components for the traits to be improved. Therefore, the collection of pertinent information that can aid in determining the value of an individual animal concerning these characteristics is crucial. To this end, the International Center for Agricultural Research in the Dry Areas (ICARDA), the International Livestock Research Institute (ILRI), the University of Natural Resources and Life Sciences, Vienna (BOKU), the National Agricultural Research System (NARS) in collaboration with farmers identified breeding objective traits for Bonga sheep producing communities.

Subsequently, in 2009, the Community-Based Breeding Program (CBBP) was launched for the breed, marking a significant milestone. The program was designed to enhance the genetic potential of Bonga sheep and improve their overall productivity. To facilitate this, a comprehensive system was established to record performance and pedigree data for the identified traits, meticulously documenting the progress within the flocks of participating farmers who willingly contributed to the initiative [7]. This enable the estimation of genetic parameters through Best Linear Unbiased Prediction (BLUP) method in the later stages. Therefore, this collaborative effort aimed to empower Bonga sheep producing communities and promote sustainable breeding practices for long-term genetic improvement.

Accurately determining and understanding reliable genetic parameters is essential for designing breeding program, understanding genetic variation, enhancing breeding efficiency and achieving genetic progress in animals. These parameters are valuable not only for evaluating the breeding value of different traits but also for optimizing breeding strategies, predicting selection outcomes, sustaining genetic diversity, and uncovering the genetic mechanisms behind quantitative traits [8]. Accordingly, there are reports on genetic parameter estimates for Bonga breed using the aforementioned data sets. For instance, [9] conducted estimations for growth traits, while [10] focused on reproductive traits of the breed. Additionally, [11] considered both growth and reproductive traits in his study. All studies employed the univariate animal model for their investigations, leading to less effective selection strategies for overall improvement since they focus on one trait. Besides, univariate model might be less precise if the trait of interest is influenced by other traits. It is apparent that precision in estimating genetic parameters plays a pivotal role in designing effective breeding programs. The accuracy of these estimates is highly contingent on the methodology and models employed for estimating (co)variance components and genetic parameters.

Recognizing the importance of precision in estimation, the use of multiple trait genetic evaluation, which exploits information on all correlated traits, offers more accurate and efficient estimates of breeding values with minimal bias compared to single trait evaluation as suggested by [12]. This accurate simultaneous estimation of genetic parameters of multiple traits ensures balanced genetic progress across all economically important traits, which directly impact the profitability of farmers. It also facilitates the simultaneous estimation of genetic parameters and breeding values for multiple traits, accounting for genetic correlations between them. These correlations reflect the extent to which the same genes influence different traits. Additionally, the model provides insights into how traits interact and how selection for one trait might affect others. It also helps in making more informed selection decisions by considering the overall genetic merit of animals across multiple traits. This stresses the significance of adopting comprehensive approaches that consider the interaction of various traits for a more robust understanding of genetic parameters in Bonga sheep.

Furthermore, enhancing ewe productivity through direct selection for reproductive traits face challenges for two primary reasons [13]. Firstly, heritability estimates for reproductive traits, as documented in the literatures, are generally low [11,14,15]. Secondly, these traits are challenging to measure at an early stage in life [13]. Consequently, the exploration of traits exhibiting moderate to high and favourable correlations with reproductive traits remains crucial. However, there is a deficiency of earlier such reports for Bonga sheep. Moreover, investigations related to survival traits were also lacking for the breed. In light of these gaps in knowledge, this study was designed with the aim of estimating covariance components, genetic parameters, and correlations of growth traits with reproductive and survival traits using a multivariate animal model.

## 2. Materials and methods

### 2.1 Description of the study area

**Fig 1:**
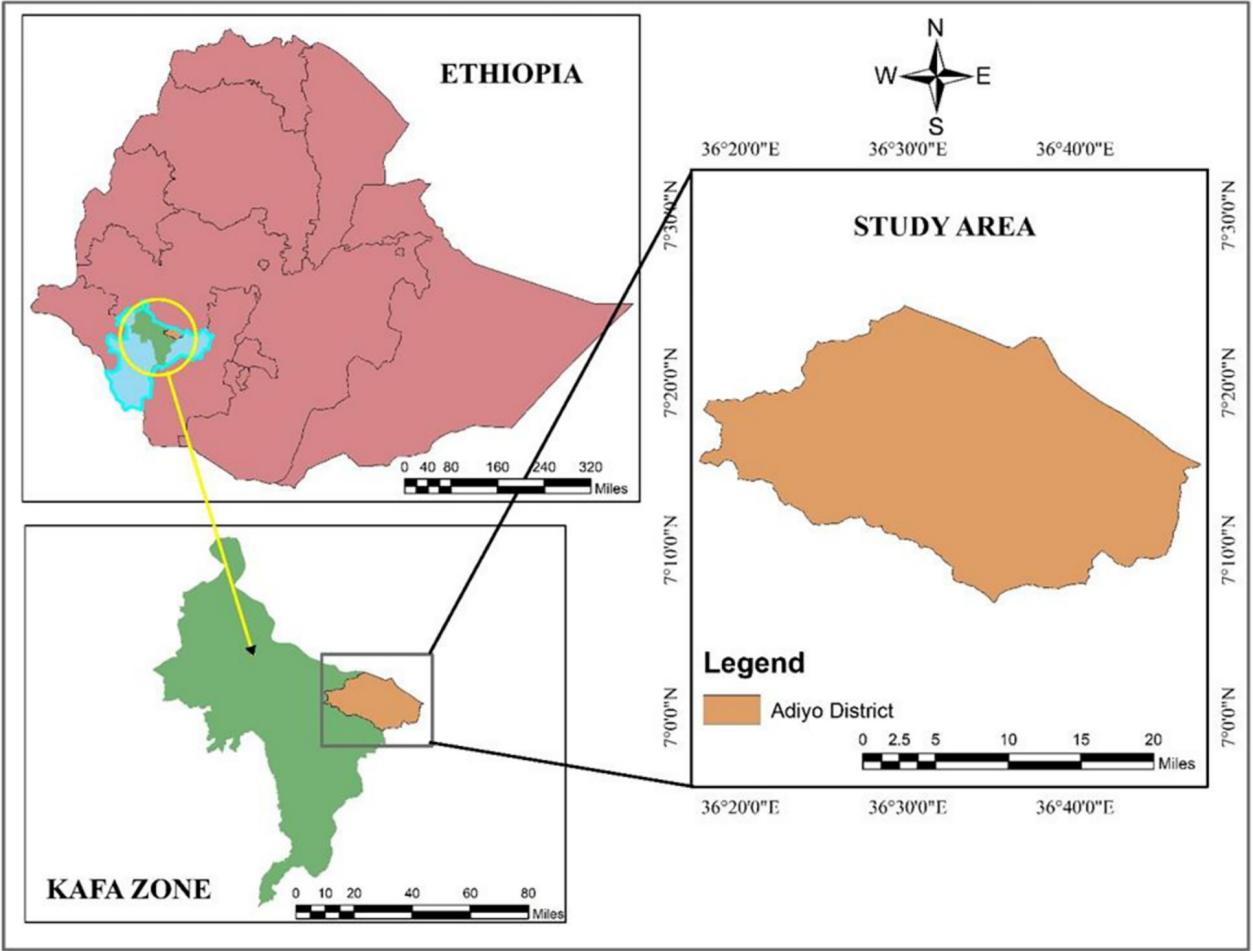
Map of the study area.

The study was conducted in the Kafa zone (Fig 1), a region where CBBP have been ongoing since 2009. Situated 460 kilometers from the national capital, Addis Ababa, the Kafa zone is located within the newly established administrative region known as the Southwest Ethiopian Peoples Regional State. Its geographical coordinates are positioned approximately 7⁰ 34′ N and 37⁰ 6′ E. The study area is distinguished by its hilly terrain, featuring an altitude range of 453 to 3361 meters above sea level, as indicated by the [16]. The region experiences a mean annual temperature of approximately 19°C, while the average annual rainfall ranges from 1500 mm in the lowlands up to 2000 mm at the highest elevations [17]. The landscape is predominantly covered by evergreen natural vegetation, as highlighted by [18]. Kafa zone has the sheep population of 424,680, out of which 69.5% are females.

### 2.2 Characteristic features of Bonga sheep

Bonga sheep is primarily found in the southwestern highlands, predominantly in Kafa zone. Its population in the main production tract is estimated to be 424,680 heads, according to the Central Statistical Agency [1]. Bonga sheep is a meat type breed known for its larger frame and heavier body weight at maturity. They are valued for their ability to grow efficiently and provide quality meat [5]. It is a prolific breed having responsible prolificacy gene of bone morphogenic protein 15 (BMP15) along with other genes controlling fertility in both sexes [19]. According to [20], it typically has a plain brown color with white or black shades, a long fat tail with a straight or twisted end, and is of the hair type. Both males and females are polled (hornless).

### 2.3 Animal management

The predominant agricultural system in the area is crop-livestock mixed production, leading farmers to keep small populations of sheep. Given the small size per household (7.8 heads) [4], the flocks of individual farmers are often merged and treated as a collective unit for the implementation of selection practices. Sheep in the region are primarily fed on natural pasture, sometimes supplemented with food leftover and improved forage such as Desho grass. During the dry season, farmers also offer their animals with the leaf of locally grown "yemogn Abeba" (*Brugmansia Suaveolens*) as part of the feeding regimen.

Lambs were weaned within three to four months, necessitating adjustments in weaning weight during data management to avoid biasness. Nearly all farmers opt for tethering their animals on their personal grazing land, employing this method as a means of controlling unwanted mating too. Thus, mating is predominantly regulated by bringing ewes in estrus to selected, improved rams maintained in the breeding programs. This approach ensures a controlled and strategic mating process within the community.

### 2.4 Data management

The data for this study were obtained from the DTREO database, developed by ICARDA in collaboration with Abacusbio and the Bonga Research Center. The data collection process involved the recruitment of enumerators from within the community who were specifically identified for this purpose. These enumerators were trained and equipped with the necessary skills to effectively collect data from the field. Reflecting data recorded at the on-farm level, the availability of sires and dams for different traits varied significantly. For example, the birth weight record comprised data from 712 sires and 3402 dams, totaling 13,269 observations. The detailed pedigree structure of Bonga sheep based on birth weight record from pooled data are indicated in Table 1. However, these numbers reduced at advanced ages. Comparable or lower records were noted for reproductive and survival traits, with varying numbers of sires and dams contributing to the dataset (Table 2).

**Table 1:** Pedigree structure of Bonga sheep for growth and reproductive traits.

Category	Growth traits	Reproductive traits
Number of record	13,269	10,726
Number of animals	15,685	12,824
Number of Sires	712	661
Number of dams	3,402	2,832
No. of animals with unknown sire	4,942	4,266
No. of animals with unknown dam	3,835	4,275
No. of animals with both parents unknown No. of sires with records & progeny in data No. of dams with records & progeny in data	2,824 539 1,159	2,693 485 910

**Table 2:** Overall least square means (SE) and effect of non-genetic factors on growth, reproductive and survival traits of Bonga sheep.

Traits	Growth			Reproductive						Survival			
Overall	BW	WW	SMW	LSB	LSW	LWB	LWW	LI	ARR		0-3m	3-6m	0-6m
N	13269	10251	6040	10400	9463	10399	9174	4491	4491		13269	12362	13269
Mean	3.5	15.7	21.8	1.33	1.18	4.5	18.6	258.1	1.97	Alive	12362	11972	11972
SE	0.01	0.03	0.05	0.01	0.01	0.02	0.08	0.64	0.01	Died	907	390	1297
CV%	18.2	19.1	17.8	36.8	42.3	33.5	41.0	16.5	43.1	SR (%)	93.2	96.8	90.3
**Effect of non-genetic factors**													
Location	**	**	**	**	**	**	**	**	**		**	**	**
Year	**	**	**	**	**	**	**	**	**		**	**	**
Season	**	**	**	*	*	*	**	na	na		**	**	NS
BT	**	**	**	na	na	**	**	**	na		**	**	**
Sex	**	**	**	na	na	**	**	na	na		*	*	**
Parity	**	**	**	**	**	**	**	**	**		*	NS	**
BWC	na	na	na	na	na	na	na	na	na		**	NS	**

N-number of observations, LSM-least square means, CV-coefficient of variation, BW-birth weight, WW-weaning weight, SMW-six months weight, LSB-litter size at birth, LSW-litter size at weaning, LWB-litter weight at birth, LWW-litter weight at weaning, AFL-age at first lambing, LI-lambing interval, ARR-annual reproductive rate, SD-standard deviation, BT-birth type, BWC-birth weight category, m-months, SR-survival rate

*-significant at *P* < 0.05

**-significant at *P* < 0.01, NS- non-significant (*P* > 0.05), na-not applicable.

The growth traits examined in this study included birth weight (BW), weaning weight (WW) which is weigh at three months age, and six-month weight (SMW). On the other hand, reproductive traits comprised lambing interval (LI), litter size at birth (LSB), litter weight at birth (LWB), litter size at weaning (LSW), litter weight at weaning (LWW), and annual reproductive rate (ARR); which was calculated as litter size * 365 days/LI (abortion and death at birth were not considered in the calculation). For survivability, lambs that survived and sold until weaning (three months of age), from weaning to six months of age, and from birth to six months of age were counted and considered censored and given a code of 1. Conversely, for lambs that died during these stages, a code of 0 was given.

The data structure, including the number of available records, arithmetic means (μ), standard error (SE), and coefficient of variation (CV) for each trait, is presented in Table 2. As mentioned earlier, weaning weights were measured at approximately three months of age. To prevent bias, adjustments were made to their weaning weight to 90 days. Similarly, the six-month body weight was adjusted to 180 days using the method outlined by [21]. The formulas for these adjusted weights are provided below:

$$AWW=\frac{90(WW-BW)}{D}+BW$$ (1)

$$ASMW=\frac{180(SMW-BW)}{D}+BW$$ (2)

Where, BW-birth weight, WW-weaning weight, SMW-six months (180 days) weight, D-number of days, AWW-adjusted weaning (three-month) weight, and ASMW-adjusted six-month weight

### 2.5 Data analysis

Preliminary analysis was conducted on the data to comprehend its structure and identify various sources of environmental variation influencing the traits under study. The GLM procedure of the Statistical Analysis System (SAS ver. 9.0) was utilized for growth and reproductive data analysis, while binary logistic regression was employed for survival data. These analysis aimed to determine the fixed factors to be incorporated into the genetic model. Non-genetic factors that had significant influence on the response variables considered were included in the later genetic analysis, which are CBBP sites: two levels (Boka and Shuta), year of birth/lambing: thirteen levels (2009–2021), season of birth/lambing: two levels (wet/April to October/ and dry/November-March/ season), birth type: two levels (singles and multiples), sex of lamb: two levels (male and female), parity seven levels (1, 2, 3, 4, 5, 6, and ≥7), and birth weight category (for survival traits): six levels (≤2, 2.1–2.5, 2.51–3, 3.1–3.5, 3.51–4, and >4kg), according to [22]. The records for each trait and fixed factors considered in the genetic model are given in Table 2. The logistic regression model used is as follows:

$$\log\left(\frac{p}{1-p}\right)=\beta 0+\beta 1X1+\beta 2X2+\beta 3X3+\beta 4X4+\beta 5X5+\beta 6X6+\varepsilon$$ (3)

where P, probability that an animal is survived; β0, intercept; X1 is the effect of location; X2 is the effect of year of birth; X3 is the effect of season of birth; X4 is the effect of birth type of lambs; X5 is the effect of sex of lamb; X6 is the effect of the birth weight category of lambs. Corresponding regression coefficients are indicated by β1- β6. ε is the residual error corresponding to responding variable. Odds can be converted to probability as follows, $p=\frac{odds}{1-odds}$ while odds or odds value is, $odds=\frac{p}{1-p}$.

The Average Information Restricted Maximum Likelihood method (AI-REML) of WOMBAT [23], fitting multivariate animal model was used to estimate covariance components, genetic parameters, and genetic correlation. The animal’s permanent environment for reproductive traits except LI and the dam’s permanent environment for growth traits were fitted in the model, along with the direct additive, maternal additive genetic, and residual effects. Wombat evaluated convergence of the analysis based on a change in log L of <5 × 10^−4^. The model for the genetic analysis for growth and reproductive traits was as follows:

$$y=Xb+Z_{1}a+Z_{2}pe_{A}+Z_{3}pe_{D}+Z_{4}m+e$$ (4)

where y is the vector of records of growth, and reproductive traits considered and b, a, pe_A_, pe_D_, m, and e are vectors of fixed, direct additive genetic, animal permanent environment, maternal permanent environment, maternal genetic, and residual effects, respectively with their association incidence matrices **X**, **Z**_1_, **Z**_2_, **Z**_3_, and **Z**_4_.

Inbreeding coefficients were compiled from WOMBAT outputs. The genetic trends were estimated by the weighted regression of the average breeding value of the animals on the year of birth.

Covariance components and genetic parameters for survival traits and their correlation with growth traits were estimated using **D**erivative-free **MU**ltivariate analysis (DMU), employing modules such as DMU1 and DMUAI for analysis. DMU1 was employed to read the driver files containing data description, model, and variance structure (prior variances and covariance). It also recoded the data file and pedigree file for use in the DMUAI module. DMUAI, in turn, was employed for estimating covariance components, genetic parameters, and correlations between growth and survival traits, applying the multi-trait threshold-linear model, which combines a linear model for growth and a threshold model for survival, with Restricted Maximum Likelihood (REML) as the typical estimation method as proposed by [24]. This setup allows DMU to estimate both the genetic and phenotypic correlations between these two traits. The analysis consisted of an examination of four traits simultaneously, encompassing three growth traits and one survival trait.

The genetic model included fixed factors (Table 2) with explanatory power on the traits, and animal additive genetic effect in addition to residual effect from random factors. The model is outlined below:

y=Xb+Za+e (5)

where y is the vector of records of survival traits considered and b, a, and e are vectors of fixed, direct additive genetic, and residual effects, respectively with their association incidence matrices denoted by **X**, and **Z**.

## 3 Results

### 3.1 The effect of fixed factors on the traits

The overall means for BW, WW, SMW, LWB, and LWW were 3.5, 15.7, 21.8, 4.5, 18.6 kg, respectively (Table 2). These values of Bonga sheep for reproductive traits such as LSB, LSW, and ARR were 1.33, 1.18, and 1.97 lambs, respectively. Explanatory variables such as location, year of birth/lambing, and season of birth/lambing significantly affected all growth traits, and most reproductive and survival traits. Cumulative survivability to six months (0-6m) was not varied with season of birth. Birth type considerably influenced the LWB, LWW, and LI in Bonga sheep. Sex of lamb also had considerable effect on composite traits, such as LWB and LWW (Table 2). All growth and reproductive traits considered in this study were significantly influenced by birth parity. Likewise, all survival traits, except post-weaning (3–6 months) survivability, exhibited significant variations with the age of dam.

The pre-weaning and post-weaning mortality rates of Bonga lambs were 6.8% and 3.2%, respectively, resulting in a cumulative mortality of 9.7% up to six months (Table 2). Birth weight category (BWC) was treated as a fixed factor only for survival traits in this study. It was observed that birth weight and survival had a direct relationship at all stages. However, it had a significant effect only on the pre-weaning and cumulative (0-6m) survivability of Bonga lambs.

### 3.2 Heritability estimates

The direct heritability for lamb traits was low to moderate, ranging from 0.15±0.03 to 0.26±0.02, with a decreasing trend as the age advanced (Table 3). The ratios of maternal permanent environmental variance to phenotypic variance (h^2^pe_D_) were 0.07±0.02, 0.25±0.01, and 0.18±0.02 for BW, WW, and SMW, respectively. Maternal additive heritability for BW, WW, and SMW were 0.14±0.01, 0.19±0.01, and 0.19±0.02, respectively.

**Table 3:** Estimates of variance components and heritability for growth, reproductive and survival traits of Bonga sheep.

Trait	σ^2^_a_	σ^2^_peD_	σ^2^_peA_	σ^2^_m_	σ^2^_p_	σ^2^_e_	h^2^_a_±SE	h^2^_peD_±SE	h^2^_peA_±SE	h^2^_m_±SE	r
Growth											
BW	0.09	0.03	-	0.05	0.35	0.18	0.26±0.02	0.07±0.02	-	0.14±0.01	-
WW	1.27	1.72	-	1.29	6.77	2.49	0.19±0.02	0.25±0.01	-	0.19±0.01	-
SMW	1.59	1.91	-	2.03	10.65	5.12	0.15±0.03	0.18±0.02	-	0.19±0.02	-
Reproductive											
LI	0.016	**-**	**-**	0.028	0.220	0.176	0.072±0.03	-	**-**	0.13±0.02	**-**
ARR	0.018	-	0.085	0.030	0.220	0.090	0.080±0.03	-	0.39±0.01	0.01±0.02	0.50
LSB	0.014	-	0.086	0.028	0.217	0.088	0.067±0.02	-	0.40±0.01	0.13±0.01	0.46
LWB	0.015	-	0.090	0.029	0.217	0.087	0.067±0.02	-	0.38±0.02	0.13±0.02	0.42
LSW	0.014	-	0.086	0.030	0.219	0.089	0.064±0.02	-	0.40±0.01	0.14±0.01	0.46
LWW	0.012	-	0.089	0.027	0.218	0.091	0.054±0.02	-	0.41±0.01	0.13±0.01	0.46
Survival											
0-3m	0.011	-		-	0.063	0.053	0.17±0.007	-	-	-	-
3-6m	0.003	-		-	0.030	0.027	0.11±0.001	-	-	-	-
0-6m	0.009	-		-	0.089	0.076	0.10±0.001	-	-	-	-

σ^2^_a −_additive variance_,_ σ^2^_pe_-permanent maternal environmental variance, σ^2^_p_-phenotypic variance, σ^2^e –residual variance_,_ h^2^a –direct heritability, h^2^_m_-maternal heritability, h^2^_peD_- dam permanent environmental variance as proportion of phenotypic variance, h^2^_peA_- animal permanent environmental variance as proportion of phenotypic variance, r—repeatability SE–standard error, m-month.

The direct heritability for dam traits was low, ranging from 0.06±0.02 for LWW to 0.08±0.03 for ARR (Table 3). In contrast, the ratio of animal permanent environmental variance to phenotypic variance was moderate to high, ranging from 0.33±0.01 to 0.45±0.03. Maternal genetic heritability for reproductive traits ranged from 0.01 to 0.14, with the lowest and highest values observed for ARR and LSW, respectively. Repeatability estimates for reproductive traits were within the range of 0.42 to 0.55. The heritability estimates for survival traits from the multivariate analysis were computed by fitting only the additive genetic effect in the model. As a result, the heritability of survivability to 3, 3–6, and to 6 months were 0.17±0.007, 0.11±0.001, and 0.10±0.001, respectively.

### 3.3 Genetic and phenotypic correlations

The additive genetic correlations between growth traits ranged from low (-0.038) to high (0.786). The lowest was observed between BW and SMW, while the highest between WW and SMW (Table 4). The phenotypic correlations were from moderate to high valued 0.284, 0.315, and 0.711 for BW-SMW, BW-WW, and WW-SMW, respectively. The ratio of both phenotypic and genetic covariances to their respective sample errors were considerably greater than 2 for all combinations of traits, suggesting the evidence of significant correlations between paired traits.

**Table 4:** Estimates of genetic and phenotypic correlations between growth, reproductive and survival traits.

Traits	r_G_			r_P_		
	BW	WW	SMW	BW	WW	SMW
**Growth**						
BW		0.150±0.078	-0.049±0.097		0.315±0.010	0.284±0.012
WW			0.778±0.052			0.711±0.007
**Reproductive**						
LI	0.653±0.140	0.601±0.145	0.442±0.168	0.003±0.002	0.039±0.017	0.037±0.020
ARR	0.609±0.136	0.577±0.142	0.451±0.163	0.009±0.003	0.044±0.017	0.042±0.020
LSB	0.488±0.105	0.440±0.118	0.458±0.127	0.042±0.010	0.113±0.013	0.146±0.016
LWB	0.487±0.105	0.439±0.118	0.456±0.127	0.042±0.010	0.113±0.013	0.146±0.016
LSW	0.550±0.108	0.451±0.120	0.379±0.136	0.044±0.010	0.114±0.013	0.161±0.016
LWW	0.572±0.121	0.455±0.133	0.351±0.152	0.034±0.010	0.103±0.013	0.146±0.016
**Survival**						
0-3m	0.002±0.001	0.414±0.020	0.537±0.025	0.001±0.000	0.633±0.017	0.775±0.023
3-6m	0.079±0.010	0.049±0.004	0.346±0.021	0.019±0.003	0.042±0.003	0.411±0.031
0-6m	-0.123±0.021	0.080±0.012	0.085±0.003	-0.016±0.010	0.064±0.012	0.197±0.032

rG—genetic correlation, r_P_- phenotypic correlation.

The genetic correlation coefficients among growth and reproductive traits were significant and predominantly positive, ranging from moderate (0.357) to very high (0.616). Birth weight demonstrated positive and moderate to high correlations with all reproductive traits, ranging from 0.487 to 0.616. The lowest was for BW and LWB, while the highest was for BW and LI. Similarly, WW showed positive genetic correlations with all reproductive traits. Its correlation with the reproductive traits varied between 0.440 and 0.590 (Table 4). Six months weight also exhibited positive genetic correlations with all reproductive traits. It ranged from 0.351 for SMW and LWW to 0.458 for SMW and LSB.

Birth weight showed positive and low genetic correlation with all survival traits except cumulative survivability (0-6m), for which it is negative. Its genetic correlation with pre-weaning survival is not significant. Weaning weight displayed a positive and low (0.049) to moderate (0.414) genetic correlation with all survival traits studied. Six months weight exhibited a positive genetic correlation with the survival traits at all stages considered. It ranged from low to moderate, with values of 0.085, 0.346, and 0.537 between SMW and up to 6, 3–6, and up to 3 months survivability, respectively.

The phenotypic correlation between growth and reproductive traits was generally low, positive, and statistically significant ranging from 0.009 to 0.161, except that the phenotypic correlation between birth weight and lambing interval was non-significant. Accordingly, all reproductive traits had low phenotypic correlations with BW, ranging from 0.009 to 0.044. Similarly, all reproductive traits exhibited positive phenotypic correlation with WW. It’s correlation with the traits ranged from 0.044 to 0.113. All reproductive traits had positive phenotypic correlation with six months weight ranging from 0.041 to 0.161. Except, between BW and cumulative survival to six months, all survival traits exhibited positive phenotypic correlation with growth traits, ranging from 0.001 to 0.775 (Table 4).

Both birth and weaning weight exhibited very low and positive phenotypic correlations with all survival traits, except for a high correlation (0.633) between weaning weight and pre-weaning survivability (Table 4). However, six months weight had positive and low to high phenotypic correlations with survival traits at all stages, ranging from 0.197 to 0.775. Insignificant phenotypic correlation was observed between BW and cumulative survival to six months (0-6m).

### 3.4 Inbreeding levels in Bonga sheep

**Fig 2:**
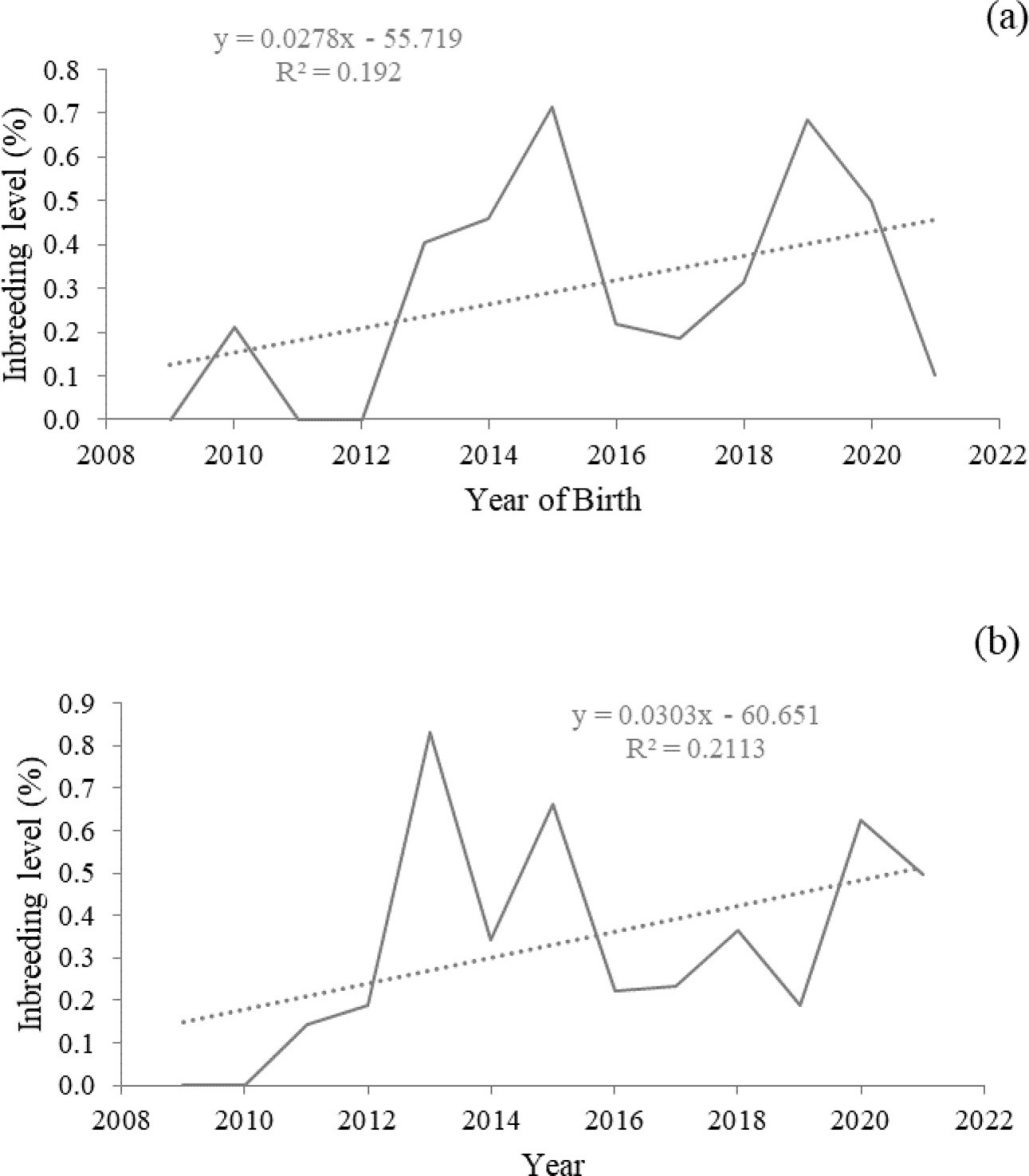
Inbreeding level of Bonga sheep in Boqa (a) and Shuta (b) sites across birth years.

The average inbreeding coefficient for Bonga sheep after 13 years (2009–2021) of selection were 0.29 and 0.33% for Boqa and Shuta sites, respectively; with annual inbreeding rate of 0.03% (p<0.001) for both sites. The level of inbreeding were slightly increased over the years in both sites. However, it remained lower than 0.5% except in the years 2015, and 2019 for Boqa and 2013, 2015, and 2020 for Shuta site (Fig 2). The average inbreeding coefficients among inbred animals were 16.5% and 15.5% for Boqa and Shuta sites, respectively.

### 3.5 The genetic trend for growth and reproductive traits

**Fig 3:**
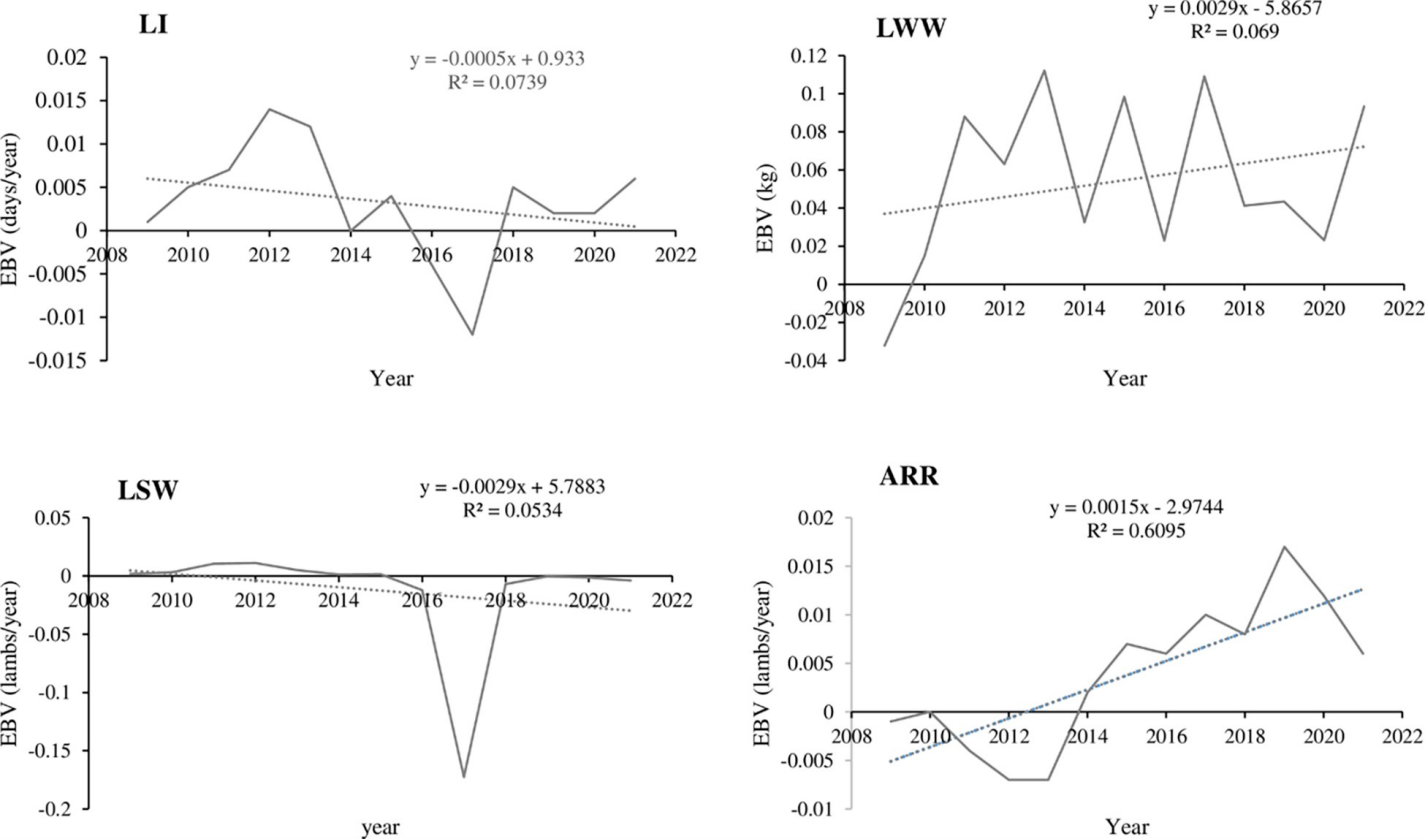
The estimated annual genetic trend of some reproductive traits from pooled data.

The genetic trend for all traits investigated were fluctuating across the selection years. The additive genetic trend obtained from multi-trait analysis of pooled data for growth traits was found to be significantly increasing (P<0.001) for WW and SMW with the rates of 0.0433 and 0.128 kg, respectively, while it was decreasing for BW. The genetic changes for reproductive traits too were highly significant with very low rate of selection response annually as depicted in the table below. Graphical genetic trend for some reproductive traits are given in Fig 3. In this study, increasing trend was observed only for ARR, LSB, and LWB among reproductive traits (Table 5).

**Table 5:** The genetic trend for growth, and reproductive traits of Bonga sheep from pooled data.

Traits	Trend	Level of significance
Birth weight (BW)	-0.0033^γ^	*
Weaning weight (WW)	0.0433 ^γ^	**
Six months weight (SMW)	0.128 ^γ^	**
Lambing interval (LI)	-0.0005^λ^	**
Annual reproductive rate (ARR)	0.0015^ε^	*
Litter size at birth (LSB)	-0.0019 ^ε^	**
Litter size at weaning (LSW)	-0.0029 ^ε^	**
Litter weight at birth (LWB)	0.0011 ^γ^	**
Litter weight at weaning (LWW)	0.0029 ^γ^	**

m—Months, NS-non-significant γ - kg/year, λ - day/year, ε - lambs/year

*-P<0.05

**—*P<0*.*001*.

**Fig 4:**
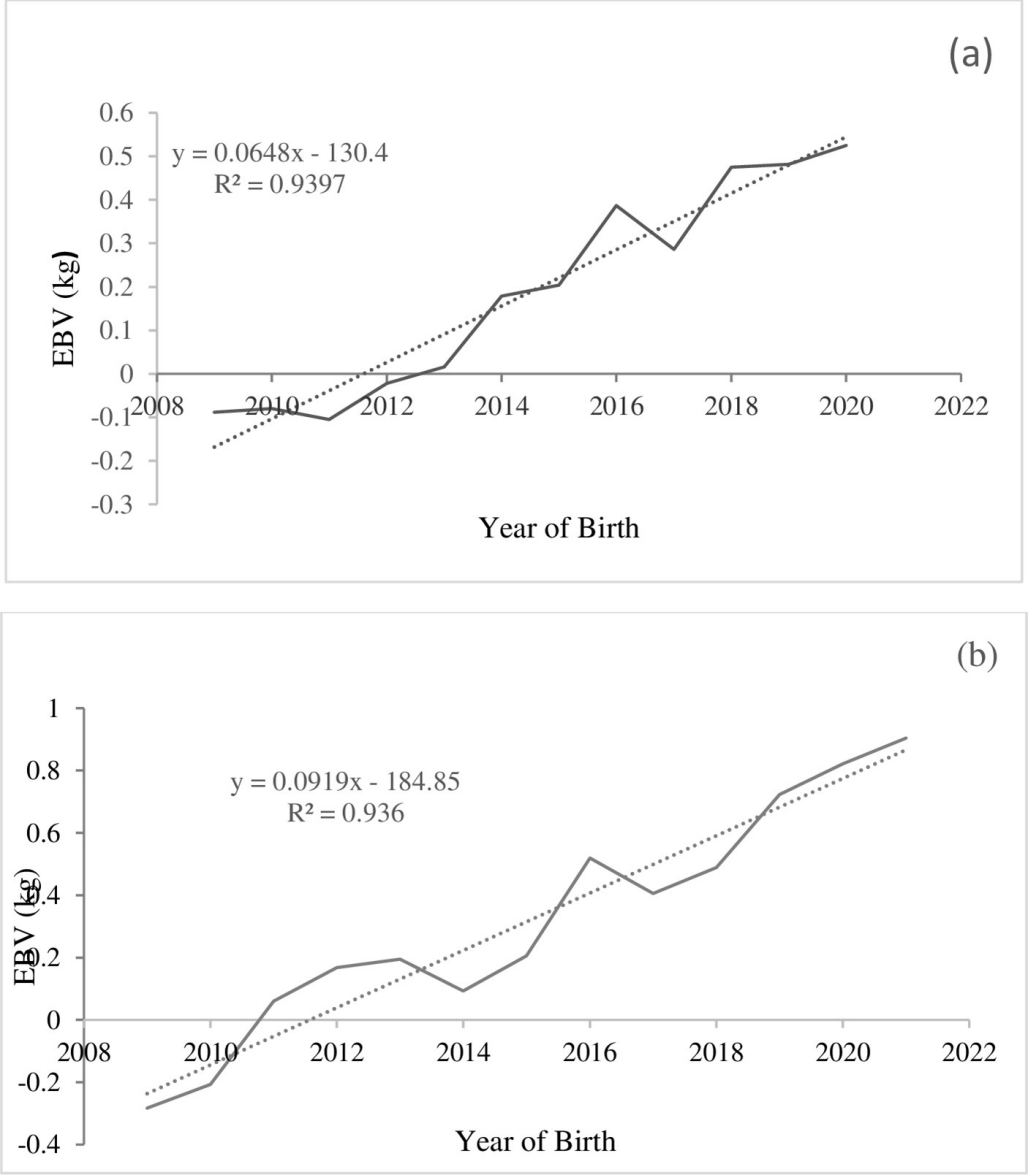
The annual additive genetic trend of six months weight for Boqa (a) and Shuta (b) sites.

The annual genetic trends for each location are provided in Table 6. In both locations, most traits showed significant progression, except for LI and ARR at the Boqa and LWB at the Shuta site (P<0.05). With the exception of BW, LI, and LSW, all traits exhibited positive progress in both sites. However, slightly better genetic trend was observed at the Shuta site compared to the Boka site for most traits, such as WW, SMW, LI, ARR, and LWW. Fig 4 indicates the annual genetic trend of six months weight for Boqa (a) and Shuta (b) sites, which is increasing with the rate of 0.065 and 0.092 kg.

**Table 6:** The annual genetic trend for growth, and reproductive traits of Bonga sheep in different locations.

Traits	Boqa		Shuta	
	Genetic trend	P-value	Genetic trend	P-value
Birth weight	-0.0024^γ^	0.001	-0.0011^γ^	0.022
Weaning weight	0.0043 ^γ^	0.001	0.0413 ^γ^	0.001
Six months weight	0.0648 ^γ^	0.001	0.0919 ^γ^	0.001
Lambing interval	-0.429^λ^	0.493	-0.5519^λ^	0.771
Annual reproductive rate	0.0008^ε^	0.210	0.0079^ε^	0.005
Litter size at birth	0.0019 ^ε^	0.001	0.0025 ^ε^	0.001
Litter size at weaning	-0.0019 ^ε^	0.001	-0.0007 ^ε^	0.001
Litter weight at birth	0.0026 ^γ^	0.001	0.0039 ^γ^	0.176
Litter weight at weaning	0.0095 ^γ^	0.001	0.0285 ^γ^	0.001

m—Months, γ - kg/year, λ - day/year, ε - lambs/year.

## 4. Discussions

The direct, maternal genetic, and variance ratio of maternal permanent environment for growth traits of Bonga sheep were moderate, except the low variance ratio of maternal permanent environment and maternal genetic heritability for BW. The moderate direct heritability for growth traits at different stages indicates the existence of genetic variability and thus, a moderate response of Bonga sheep to within breed selection. Several previous studies in sheep, including those conducted by [25–28], have also reported similar findings of moderate direct heritability for growth traits.

Similarly, the moderate heritability of maternal genetic and variance ratio of maternal permanent environment for growth traits also indicates their significant contribution to the phenotypic variance of the breed. This is in accordance with the reports of [29–31], for different breeds. Therefore, considering these random factors during the estimation of breeding values is crucial for Bonga sheep. [32,33] reported the variance ratio of maternal permanent environment of 0.20 and 0.24, respectively for Malpura sheep, which is higher than the current finding. The effects of maternal environment were significant at birth, reflecting variances in the uterine environment, as well as the quality and capacity of the uterine space for fetus [34]. Contrarily, the variance ratio of maternal environment for BW was low, which might be an indication for lower variability among Bonga ewes with regard to uterine environment.

The low direct heritability for the investigated reproductive traits shows the limited importance of additive effects to these traits in the breed, indicating sluggish genetic progress of the traits through selection. Conversely, the ratio of animal permanent environmental variance to phenotypic variance ranged from moderate (0.33) to high (0.45), emphasizing the stronger influence of environmental factors on these traits. Therefore, minimizing the impact of environmental factors and adopting improved management practices are more valuable intervention strategies for enhancing reproductive performance in Bonga sheep, in addition to selection. The low direct heritability for reproductive traits in this study is consistent with the findings reported in different literatures [9–10,35,36].

Studies reported low direct heritability for survival traits at different stages [37–42]. Gaur [43] also reported low direct heritability of survival to weaning (0.04) and up to six months (0.07) for Harnali sheep. All these reports are in line with our results. The results suggest that the additive genetic effect on these traits in the breed is minimal. Consequently, variations in these traits are largely attributed to non-additive genetic components of animals (dominance and epistasis), environmental factors and management practices. Similar to our results, numerous environmental factors, including dam parity, year and season of birth, lamb birth weight, stock management influence lamb survival, which could explain why estimates for the heritability of lamb survival are low [44]. Given that the animals were kept under farmer conditions, these outcomes are expected. Thus, the present findings suggest that lamb survival could be improved through better management practices. The importance of improving management practice to increase survivability has been reported [39].

The repeatability estimates obtained currently are more or less comparable with the findings of [11] for LI, LS, and ARR for the same breed in another locations. However, [10,45,46] reported lower estimates than the current magnitudes LI and LS for Bonga, Horro, and Pelibuey ewes, respectively. However, the repeatability estimates for the traits are much higher than the direct heritability, indicating more influence of environmental factors and non-additive effects.

The low genetic correlations for BW-WW, and BW-SMW, imply that there was no correlated response while selecting for SMW in Bonga sheep; which is desirable via avoiding lamb losses due to difficulty during lamb delivery. This concurs with the results of Haile et al (2020) for Bonga and Horro sheep and [42] for Washara sheep. However, [27,47,48] reported higher magnitudes for the same combinations of traits for Iranian Karakul, Corriedale and Doyogena sheep, respectively. This might be due to differences in the level of intervention, selection intensity, or the available pedigree structure available for different flocks. On the other hand, the negative genetic correlation between birth weight and six-month weight might be due to the smaller sample size for six-month weight compared to birth weight. This is evident from the larger standard error than its estimate. Contrariwise, WW and SMW were highly correlated, suggesting that they might had experienced higher effect of pleotropic loci or linkage disequilibrium. So, selection for one of these traits had favourable effect on the other and thus, selection for WW could be more preferable as it is expressed earlier in life considering its moderate direct heritability and correlation with other traits. Higher genetic correlation for the same pair of traits have been reported in the literatures [27,28,49].

The positive and moderate to high genetic correlations of BW with reproductive traits suggests that selecting for improving BW would likely yield a high genetic response in traits such as LI, ARR, LSB, LSW, LWB, and LWW. However, it’s important to note that selection based on BW may have unintended consequences, potentially leading to lamb losses due to dystocia, especially if it surpasses a certain threshold under on-farm conditions. Dystocia is mentioned as a series problem in the area in previous reports [50]. Our findings align with those of [14,51], who reported positive and higher genetic correlations between birth weight and LWB, as well as LWW, in Lori-Bakhtiari and Zandi sheep, respectively. Interestingly, the same authors noted positive but lower genetic correlations between BW and LS at birth and at weaning, which differ from our current results. In contrary to our results, negative and very low BW and LI genetic correlation (−0.06) was reported for Morada Nova Sheep [52].

Similarly, the positive and moderate to high additive genetic correlations of WW with all reproductive traits, indicated that, selecting for WW would likely result in a higher indirect response in most reproductive traits. However, it’s important to realize that a positive genetic correlation doesn’t always imply a favorable relationship. This is evident in the case of WW-LI, where the expectation is a decrease in LI while increasing WW. Therefore, simultaneous improvement in both traits is minimal for Bonga sheep. In line to our results, [25,51] reported moderate to high genetic correlations between WW and litter traits, ranging from 0.21 to 0.67 for Makooei sheep and 0.37 to 0.96 for Zandi sheep, respectively. In agreement to ours, [52] reported positive but lower genetic correlation (0.15) between WW and LI than the current finding for Morada Nova Sheep.

The positive and moderate to high genetic correlation of SMW with all reproductive traits (LI, LSB, LSW, LWB, and LWW) is an indication for a higher indirect response due to selection for six months weight in litter traits. However, it’s essential to note that an undesirable response in LI may occur while selecting to improve SMW. Vatankhah and Talebi [14,51] reported lower correlations for litter size and higher correlations for litter weight traits with SMW in Lori-Bakhtiari and Zandi sheep, respectively. To the author’s knowledge, we could not find reports in the literature regarding the correlations of six months weight with LI and ARR. Positive and very weak to weak phenotypic correlations were realized between growth and reproductive traits. In agreement to the current study, [14,25] indicated very low phenotypic correlation of litter traits with growth traits.

Generally, any breeding program tends to improve survival and growth performance of lambs along with reproduction, to improve overall production of the flock. In the current study, the genetic correlation (r_G_) between growth and survival traits were low to moderate, while the corresponding phenotypic values were from low to high. Birth weight exhibited negative and moderate additive genetic correlations with pre-weaning survival in Washera sheep [42] and sheep farms in New Zealand [53], respectively; which is different and much higher than our results. The positive genetic correlation between WW and lamb survival traits (Table 3) in our result implies that selecting for heavier Bonga lambs at three months of age is likely to promote survival of lambs at all stages. Similarly, positive and comparable (0.46) correlation between WW and pre-weaning survivability has been reported Washara sheep [42].

Lamb survival and six months body weight are among the breeding goal/selection traits of Bonga CBBPs. According to [54], selection on multiple traits simultaneously can be applied for traits that are both in the breeding goal. In the current study, both the genetic and phenotypic correlations between SMW (the main selection trait in Bonga CBBPs) and survival traits were positive (Table 3). Therefore, survivability increased with an increase in SMW, indicating possibility of joint improvement through selection for only one of these traits in Bonga sheep. Likewise, [42] reported positive genetic correlation and comparable magnitude (0.5) to ours for SMW and pre-weaning survival for washara sheep. Generally, there are no much reports on combination of growth and survival traits in literatures.

The coefficient of inbreeding found from pooled data is marginally higher than 0.34% reported by [9] for the same breed, indicating a slightly increasing trend of inbreeding over years. The increasing trend in the current study is in line with the finding of [48] for Doygena sheep. However, the inbreeding coefficient from pooled data and that of specific location are still far lower than the critical level (6.25%) reported by [55]. Both higher and lower inbreeding coefficients than the current finding are found in literatures. For instance, [56] reported 1.7 for Menz sheep under on-station condition, while 0.30 was reported for Doyogena sheep [48]. Therefore, the fitness and performance of Bonga sheep is not threatened due to inbreeding effect at least for now. This is mainly due to larger pooled flock for selection, rotation of breeding rams between ram use groups, and culling of breeding rams after only two years of mating service in the Bonga CBBPs. Nevertheless, care should be taken to maintain the inbreeding coefficient to the lower possible level; so that the genetic variability of the breed is conserved.

The genetic trend is often considered as an indicator of the direction of change across generations [54]. However, Fig 2A and 2B depicted the genetic trend for six months weight of Bonga sheep expressed per birth year due to overlapping generations in the village flock. In the current study, the genetic trend for all investigated traits deviated from the expected linearity, exhibiting a fluctuating pattern. This fluctuation might be attributed to inconsistent feed availability due to continually changing environmental conditions.

The annual additive genetic progress was highly significant (P<0.001) for most traits. Consistent with our results, [57] reported a negative and significant genetic trend in BW for Sardi sheep. Similarly, negative but insignificant genetic progress in BW have been reported for Bonga and Horro sheep [9] and for Doyogena sheep [48]. Unlike to our results, the above authors used a univariate animal model for the estimation of breeding values, which might have significant role for the observed alterations. However, the same authors reported a positive genetic trend for WW and SMW, which aligns with our current findings. Therefore, selection for either WW or SMW would lead to the desired success. Nevertheless, the current genetic gain for SMW are (both pooled and location specific) less than that reported by [9] for the same breed. Accuracy in pedigree and phenotype recording, accuracy and alteration in selection intensity and genotype by environment interaction in the reliably changing environment might be the possible reasons for the decline at present. Climate variability which resulted in decline of forest cover has been reported in the area [58].

The estimates of annual genetic trends obtained from multi-trait analysis were positive except for BW, LI and LSW, and the magnitudes are close to zero for most reproductive traits, which can be attributed to the low additive heritability estimates of these traits. Aguirre [59,60] reported negative and very small annual genetic trend of LS for Santa ines (-0.0003) and Bonga (-0.0002) sheep, respectively, which are different from the current finding. Similarly, positive but higher genetic trends than the current magnitudes have been reported in LSB, and LWB for Iran-Black sheep [61]. Haile [7] reported insignificant but positive trends for litter weight traits and a negative trend for LI in Awassi sheep. With regard to the specific location, for more than half of the traits considered in the study, the annual genetic progress for the Boqa site is slightly lower as compared to Shuta. Possible reasons for this disparity might include inaccuracies in data recording and inefficient ram utilization. Therefore, exhaustive follow-up of the Boqa site should be given due attention.

## 5. Conclusion

The heritability estimates obtained revealed that there are genetic variabilities for growth traits in Bonga sheep, which can be exploited through direct or indirect selection for further improvement in the community’s flocks. Conversely, environmental influences had a more pronounced impact on reproductive and survival traits than genetic effects. Therefore, minimizing environmental effects through modifications to management practices is crucial for improving these traits along with selection.

The wide-ranging genetic correlations observed between traits suggest the presence of one or more pleiotropic gene loci and/or the influence of linkage disequilibrium in Bonga sheep. The estimates of genetic correlations between growth traits and reproductive and survival traits were positive and ranged from low to high, suggesting that selection based on improving weaning weight or weight at six months of age may enhance genetic merit in ewe productivity and lamb survival. This highlights the importance of considering both growth and reproductive/survival traits (especially LWW) in breeding programs to achieve optimal outcomes in sheep production. Optimizing the breeding program by adding litter weight at weaning to the selection index is advisable for making balanced selection decisions, as this trait encompasses maternal traits and abilities.
